# Efficacy of ceftazidime in a murine model following a lethal aerosol exposure to *Burkholderia pseudomallei*

**DOI:** 10.1038/s41598-023-31131-8

**Published:** 2023-03-10

**Authors:** Denise A. Pfefferle, Michael Hackett, Michael S. Anderson, Seth Gibbs, Lisa N. Henning, April C. Joice, Gabriel T. Meister

**Affiliations:** 1 Battelle Memorial Institute, Columbus, OH USA; 2 AmplifyBio, West Jefferson, OH USA

**Keywords:** Animal disease models, Antibiotics, Bacterial infection

## Abstract

Melioidosis is an endemic disease in numerous tropical regions. Additionally, the bacterium that causes melioidosis, *Burkholderia pseudomallei*, has potential to be used as a biological weapon. Therefore, development of effective and affordable medical countermeasures to serve regions affected by the disease and to have medical countermeasures available in the event of a bioterrorism attack remains critical. The current study evaluated the efficacy of eight distinct acute phase ceftazidime treatment regimens administered therapeutically in the murine model. At the conclusion of the treatment period, survival rates were significantly greater in several of the treated groups when compared to the control group. Pharmacokinetics of a single dose of ceftazidime were examined at 150 mg/kg, 300 mg/kg, and 600 mg/kg and were compared to an intravenous clinical dose administered at 2000 mg every eight hours. The clinical dose has an estimated 100% *f*T > 4*MIC which exceeded the highest murine dose of 300 mg/kg every six hours at 87.2% *f*T > 4*MIC. Based upon survival at the end of the treatment regimen and supplemented by pharmacokinetic modeling, a daily dose of 1200 mg/kg of ceftazidime, administered every 6 h at 300 mg/kg, provides protection in the acute phase of inhalation melioidosis in the murine model.

## Introduction

*Burkholderia pseudomallei* is the causative agent of the disease melioidosis. Melioidosis is endemic in Southeast Asia, Northern Australia^1^, and other tropical regions across the globe^2^. *B. pseudomallei* remains a United States Centers for Disease Control (CDC) Tier 1 overlap select agent as it presents a notable risk of deliberate misuse and poses a severe threat to both human and animal health^3^. While several treatment protocols for melioidosis are available, outcomes vary and *B. pseudomallei* is intrinsically resistant to several classes of antimicrobials^4,5^. Therefore, there remains a need to discover novel efficacious and cost-effective medical countermeasures (MCMs) which eradicate the body of *B. pseudomallei* following infection. Currently, biphasic treatment is employed for melioidosis, where the acute phase consists of intensive intravenous therapy for at least ten days and the second phase consists of oral eradication therapy in duration of at least 12 weeks^6^. Acute phase treatments include administering ceftazidime, meropenem, or imipenem intravenously while eradication phase treatments include administering co-trimoxazole or co-amoxiclav orally^6,7^. In 1986, ceftazidime was first investigated for the treatment of melioidosis in Thailand where the standard of care acute phase treatment at that time carried a mortality rate ranging from 38 to 61%^7^. Overall mortality rates have fallen with the use of ceftazidime; therefore, the third generation cephalosporin remains a standard of care antibiotic for the disease^8^. The PK/PD driver for cephalosporins such as ceftazidime is *f*T > MIC. *f*T > MIC targets between 30 and 40% in the neutropenic thigh model of other pathogens have been associated with bacteriostasis^9–11^. Through in-vitro characterization, Mouton et al*.* confirmed *f*T > MIC between 35 and 38% would produce a static effect in mice administered ceftazidime following *Pseudomas aeruginosa* infection^12^. The optimal clinical target for *P. aeruginosa* has been reported as 100% *f*T > 4*MIC; however, this target was not readily applicable to severe infections of *B. pseudomallei* as it did not prevent mortality in most subjects^13^. While ceftazidime is a viable option for acute phase treatment, *B. pseudomallei* is a facultative intracellular pathogen that can evade host clearance mechanisms, thus requiring the need for long term eradication therapy^14^. A major cause of relapse has been linked to eradication therapy in duration of less than 12 weeks^14^, leaving room for improvement in current treatment protocols. Several intervention targets for treating *B. pseudomallei* infection have been studied^4^ which could allow for the identification of novel or repurposed therapeutic agents to be used for the treatment of melioidosis.

The mouse is an established model of melioidosis as several strains of mice have shown to be susceptible to multiple strains of *B. pseudomallei* and to multiple routes of infection^15–23^. Additionally, several of the primary organs of infection (including the liver, lung, and spleen) are consistent with clinical melioidosis^24^. The available literature illustrates that the BALB/c mouse recapitulates the acute melioidosis disease state and is applicable for studying acute phase melioidosis therapies. Several laboratories have investigated the efficacy of antibiotics and other therapeutic agents, including ceftazidime, in the murine melioidosis model^25–32^. However, regarding ceftazidime administration, the antibiotic dosage, administration route, therapy frequency, and therapy duration have not been consistent. The route of infection, *B. pseudomallei* strain, and infective dose have also varied amongst MCM efficacy studies. This study aimed to further characterize the acute murine melioidosis model with the purpose of establishing a clinically relevant ceftazidime dose and regimen to be used as a positive control comparator in future MCM evaluations of severe infection. Through PK/PD modeling, it was determined that the clinical pharmacokinetics of ceftazidime could not be readily recapitulated in mice; therefore, target ceftazidime exposures were chosen that would result in increasing percentages of *f*T > 4*MIC with the highest target intended to approach 100%. Eight ceftazidime treatment regimens were evaluated during the acute phase of disease in the BALB/c mouse. In addition, pharmacokinetics of a single dose of ceftazidime were examined at 150 mg/kg, 300 mg/kg, and 600 mg/kg and were compared to an intravenous clinical dose administered at 2000 mg every eight hours.

A 1200 mg/kg/day dose of ceftazidime administered approximately every six hours at 300 mg/kg over 14 days provides protection to BALB/c mice through the acute phase of melioidosis but does not prevent recrudescence. In evaluations of novel MCMs for severe infections, this is an adequate acute phase dose to utilize as a comparator antibiotic or it could potentially be used to evaluate ceftazidime as part of a novel combination therapy.

## Materials and methods

### Study design

Female mice placed on study were at least 6 weeks of age at the time of arrival, weighed between 16 and 20.1 g on the day of exposure, and were free from clinical signs of disease at the time of exposure. Mice were randomized to study groups by body weight. Patterned assignment was utilized to select animals in Groups 1, 11, 12, and 13 to subgroups and to select all study animals to a challenge run order. At least one animal from each study group was selected for each challenge exposure run. One hundred ninety female BALB/c mice were exposed to aerosolized *B. pseudomallei* (strain K96243) at a targeted 8100 cfu/animal, equivalent to 50 × LD_50_ (actual mean inhaled dose of 11,100 cfu; 69 × LD_50_) on a single day. Ten aerosol exposure runs of 19 mice per run were conducted. Aerosol concentrations of all exposure runs ranged from 7450 cfu/animal to 18,300 cfu/animal (standard deviation 4119 cfu/animal). The mass median aerodynamic diameter of inhaled particles ranged from 1.26 to 1.31 µm (geometric standard deviation 1.47–1.56 µm), allowing for lower lung deposition of the inhaled particles^33,34^. Following aerosol exposure, mice were treated with test (ceftazidime) or control (sterile saline) article. Two treatment cohorts were included in the study. The first cohort examined the efficacy of a 14 day ceftazidime regimen through assessment of bacterial tissue burden and survival 60 days post-cessation of treatment. Eight distinct treatment regimens were assessed (Table 1) which were comprised of two treatment frequencies: twice daily (BID) and four times daily (QID); two initiation time points: 24 h post-exposure (24 h) and 48 h post-exposure (48 h); and two dosages: 600 mg/kg/day and 1200 mg/kg/day. The 600 mg/kg/day dosage was achieved by administering 300 mg/kg BID or 150 mg/kg QID while the 1200 mg/kg/day dosage was achieved by administering 600 mg/kg BID or 300 mg/kg QID. Doses were administered approximately every 12 h for BID dosing and approximately every 6 h for QID dosing. Tissue burden was examined in the kidney, liver, lung, and spleen in all treated mice, as well as in a cohort of untreated mice at 24, 36, 48, and 60 h post-exposure to assess disease progression. A satellite cohort of mice was used for pharmacokinetic analysis of three dosages of ceftazidime following a single dose administered approximately 24 h post-exposure. No exposed animals were excluded from survival analysis. No blinding or masking was utilized during the conduct of the study.

**Table 1:** Study design—efficacy and PK cohorts.

Group	Number of animals	Cohort	Test Article	Dosage (mg/kg/day)	Dosage (mg/kg/dose)	Treatment initiation	Dose frequency
1a	5	Tissue Burden^	NA	NA	NA	NA	NA
1b	5						
1c	5						
1d	5						
2—Saline	10	Efficacy	0.9% Sterile Saline	NA	NA	24 h PC	BID × 14 days
3—24 h Low BID	10		Ceftazidime	600	300		
4—24 h High BID	10			1200	600		
5—48 h Low BID	12			600	300	48 h PC	
6—48 h High BID	12			1200	600		
7—24 h Low QID	10			600	150	24 h PC	QID × 14 days
8—24 h High QID	10			1200	300		
9—48 h Low QID	12			600	150	48 h PC	
10—48 h High QID	12			1200	300		
11a–f	24	PK*	Ceftazidime	NA	150	24 h PC	Single dose
12a–f	24				300		
13a–f	24				600		

The experimental design. Disease progression was assessed in infected, untreated animals from 24 through 60 h post-exposure. The efficacy of eight distinct ceftazidime dosing regimens was assessed in Groups 2 through 10 and included a saline control group. Pharmacokinetics of the three dosages of ceftazidime administered in the efficacy cohort were assessed in Groups 11 through 13, following a single dose of ceftazidime that was administered approximately 24 h post-exposure.

*BID* twice daily, *h* hours, *IP* intraperitoneal, *NA* not applicable, *PC* post-exposure, *QID* four times daily.

^Up to 5 mice were humanely terminated at 24 (1a), 36 (1b), 48 (1c), or 60 (1d) hours post-exposure for bacteremia and tissue bacterial burden assessment.

*Four mice from each subgroup underwent terminal whole blood collection following a single dose of ceftazidime at one of the following time points post dose: 0.25 h, 0.5 h, 1 h, 1.5 h, 2 h, or 4 h.

### Test system

Female BALB/c mice, six to eight weeks of age at arrival, were procured from Charles River Laboratories (Stone Ridge, NY). General procedures for animal care and housing met AAALAC International recommendations, current requirements stated in the “Guide for the Care and Use of Laboratory Animals” (National Research Council, Current Edition), current requirements as stated by the U.S. Department of Agriculture through the Animal Welfare Act, as amended, and conformed to testing facility standard operating procedures. The animal use protocol was approved by the Institutional Animal Care and Use Committee (IACUC) and by the United States Army’s Animal Care and Use Review Office (ACURO). Study findings have been reported in accordance with ARRIVE guidelines.

### Test and control articles

Ceftazidime for injection, USP (Hospira, Lake Forest, IL) was diluted to 95 mg/mL in sterile water for injection, USP. Dosing solutions were further diluted in sterile saline, USP to 15, 30, or 60 mg/mL to achieve dosages of 150 mg/kg/dose, 300 mg/kg/dose, and 600 mg/kg/dose, respectively. Sterile saline was administered to animals in the control group. Doses were administered intraperitoneally (IP) at a target volume of 10 mL/kg. Injections were administered at contralateral sites at each administration to minimize localized tissue damage.

### Challenge agent and aerosol exposure

*Burkholderia pseudomallei* strain K92643 was utilized for animal exposures. Strain selection was based upon previous internal characterization. Internally conducted minimum inhibitory concentration (MIC) testing of the K96243 strain yielded a MIC of 1 µg/mL for ceftazidime. The lot used for the aerosol exposures was propagated and characterized by the Battelle Biomedical Research Center. Fresh *B. pseudomallei* suspensions were prepared by inoculating stock material with LB broth with 4% glycerol (LBG) and incubating at 37 °C (± 2 °C) and shaking at 250 rpm for 18–24 h. The resulting starter culture was diluted in sterile LBG to reach an OD_600_ of 0.200 (± 0.05). This culture was then incubated at 37 °C (± 2 °C) and 250 rpm for 18–20 h. The culture was examined for purity following gram-staining. The culture was then centrifuged at 10,000 rcf for ten minutes. The resulting pellet was washed and resuspended in phosphate buffered saline with gelatin and trehalose (BSGT). The wash and resuspension procedure was repeated a total of two times. The suspension was removed and adjusted with buffer to reach an OD_600_ of 2.1 (± 0.1). Aerosol exposures were conducted as previously described^35^. An inhaled dose of 8100 cfu equating to 50 × LD_50_, was targeted for consistency with previous internal model development experiments.

### Whole blood collection and pharmacokinetic analysis

Cardiac blood collection was performed as a terminal procedure. Prior to collection, mice were anesthetized with a mixture of ketamine (80–100 mg/kg) and xylazine (5–10 mg/kg). Bacteremia specimens were collected into K_3_EDTA tubes and maintained at room temperature until processing. Pharmacokinetic (PK) samples were collected into K_3_EDTA tubes and stored on wet ice until processing. Plasma was harvested and sterile filtered using a 0.2 micron PES filter. Samples were stored in a freezer set to maintain − 80 °C until analysis.

Ceftazidime was extracted from plasma and quantitated using a high performance liquid chromatography tandem mass spectrometry (LC–MS/MS) method developed internally. It is a sensitive and high throughput method utilizing protein precipitation extraction followed by strong cation exchange (SCX) LC–MS/MS. Detection was accomplished using a Shimadzu Prominence XR Series HPLC and AB Sciex Triple Quad 5500 mass spectrometer in positive ionization mode (ESI^+^). Chromatographic separation was obtained using an Agilent Zorbax 300-SCX 5µ, 2.1 × 150 mm column, a gradient mobile phase consisting of 95:5 25 mM ammonium formate:acetonitrile (v:v) (A) and 70:30 25 mM ammonium formate:acetonitrile with addition of 500 mM ammonium formate (B) at a flow rate of 0.5 mL/min., and a column oven temperature of 30 °C. Ceftazidime pentahydrate obtained from US Pharmacopeia (USP) was used in the preparation of the solutions for the standards and quality control samples prepared in mouse plasma. A chemical analog, cefepime hydrochloride obtained from USP, was prepared as an internal standard. The addition of the internal standard allowed for reproducible quantification as the ceftazidime and cefepime were monitored using multiple reaction monitoring (MRM) at 547.0/468.0 and 481.0/86.0 respectively. Regression analysis was evaluated by analyzing a set of calibration standards at eight concentrations. The lower limit of quantitation for ceftazidime was determined to be 50 ng/mL in plasma using a 100 µL sample size.

Free plasma concentrations were adjusted using an estimated 26% and 15% protein binding for mice and humans, respectively^36^. PK and exposure parameters were estimated using non-compartmental analysis with Phoenix WinNonlin software (Certara L.P., Princeton, NJ). The following acceptance criteria were used to evaluate the concentration–time profiles: (1) the coefficient of determination (r^2^) for the terminal linear phase was greater than or equal to 0.85, (2) the time of the last observed concentration was greater than three times the half-life, and (3) AUC_∞_ had less than 20 percent of the area extrapolated. The mouse data were then fit to a compartmental model in WinNonlin which was used to simulate concentrations for PK/PD target attainment.

### Tissue collection and bacterial enumeration

At the time of necropsy, a portion of the kidney, liver, lung, and spleen were aseptically collected from each mouse, weighed, and individually homogenized in 1 mL of Dulbecco’s phosphate buffered saline with 0.01% gelatin (BSG) using a gentleMACS Dissociator (Miltenyi Biotec, Bergisch Gladbach, Germany). Tissue homogenates were serially diluted in triplicate and spread on LB agar with 4% glycerol (LBGA) or Columbia blood agar. Spread plates were placed in an incubator set to maintain 37 °C for an average of 58 h (41.4–93.1 h). Plates were enumerated and the cfu/gram of tissue calculated for statistical analysis.

### Statistical analysis

Statistical analyses were performed using SAS (version 9.4) and R (version 3.6.3) on the 64-bit platform. For determination of group sizes, power analysis based upon a single one-sided Boschloo’s Test conducted at a Type I error rate of 5% was conducted to detect statistically significant differences in survival rates between a single treated group and a control group. When assuming a control group has a 5% survival rate, ten animals per treatment group allows for 88% power when assuming the probability of survival in the treated group is at least 60%. Two additional animals included in the 48 h post-exposure groups allowed statistical significance to be maintained in the event of early deaths. The proportion of surviving animals and exact 95% confidence interval were calculated for each group in the efficacy cohort. One-sided Boschloo’s tests were used to compare survival proportions between each of the treated groups and the saline control group (Group 2) at end of study, end of treatment, and 30 days post cessation of treatment. Two-sided Boschloo’s tests were used to compare survival proportions between each pair of timepoints within each group. The Bonferroni-Holm multiple comparison procedure was used to control the overall Type I error rate across each set of tests at 5%. The log-rank test was used to test for significant differences in time to death between each of the treated groups and the saline control group. The Bonferroni-Holm multiple comparison procedure was used to control the overall Type I error rate across all the tests at 5%. Kaplan–Meier curves were plotted for each group to represent survival and time to death data visually. For quantitative assessment of bacterial loads in tissues, geometric means and 95% confidence intervals were calculated for each tissue type by group. Analysis of variance (ANOVA) models were fitted to the base-10 log-transformed bacterial loads to evaluate the effects of treatment on tissue bacterial loads. The mean bacterial load for each of the treatment groups were statistically compared to that of the saline control group (Group 2) within the model for each tissue type. Dunnett’s multiple comparison procedure was used to control the overall Type I error rate across all the tests per tissue at 5%.

## Results

### Efficacy of ceftazidime up to 60 days post-cessation of treatment

**Figure 1:**
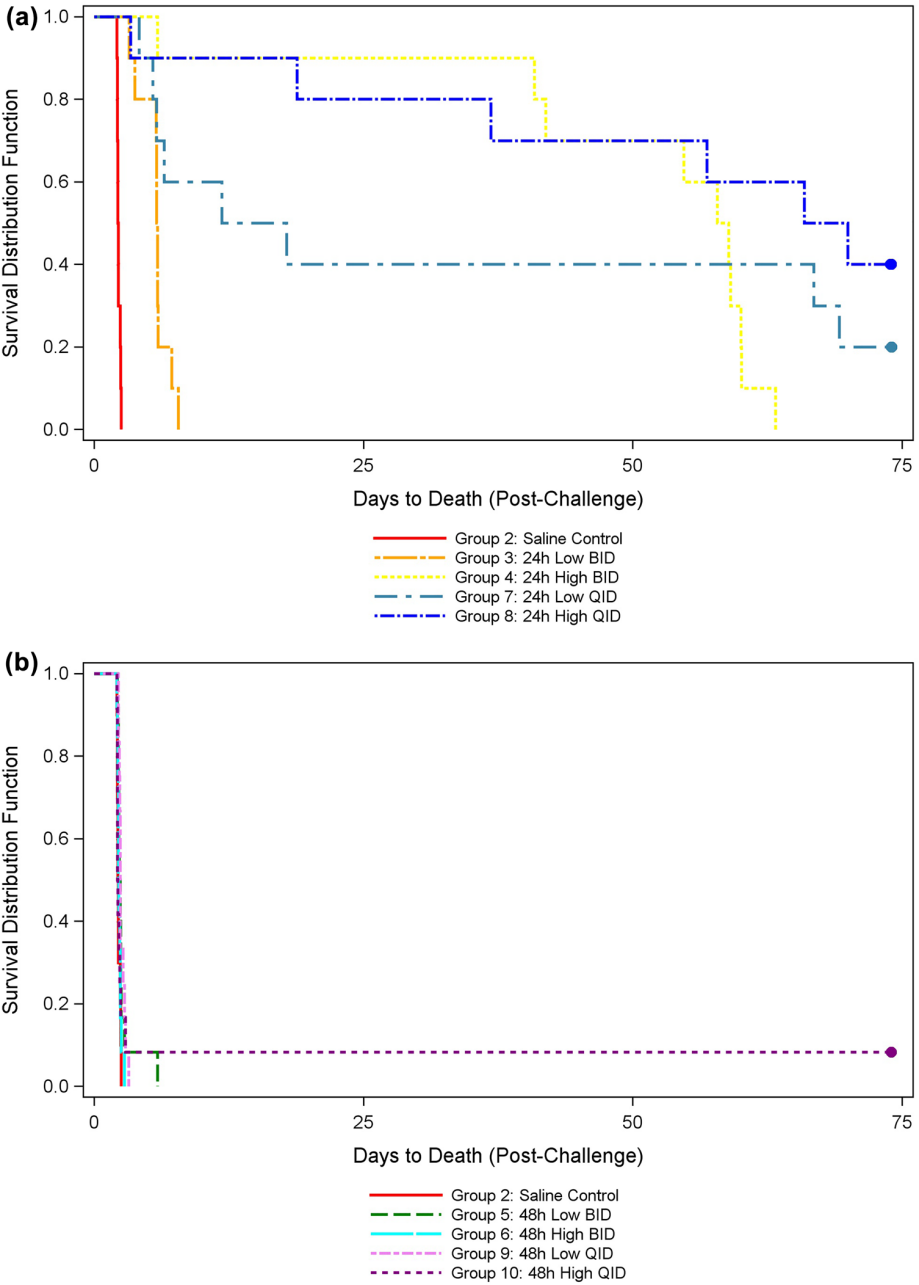
(**a**) Kaplan–Meier survival plot for 24-h intervention groups. Time to death in days for the control group and 24 h post-exposure ceftazidime treated groups (IP administration). Treatments were administered twice daily (BID) or four times daily (QID). BID low dose = 300 mg/kg/treatment, totaling 600 mg/kg/day. BID high dose = 600 mg/kg/treatment, totaling 1200 mg/kg/day. QID low dose = 150 mg/kg/treatment, totaling 600 mg/kg/day. QID high dose = 300 mg/kg/treatment, totaling 1200 mg/kg/day. Survival rates in Groups 4, 7, and 8 at the end of treatment were significantly greater than the control group (p = 0.0002, 0.0383, and 0.002, respectively). Survival rates in Groups 4 and 8 at Study Day 45 (30 days post-cessation of treatment) were significantly greater than the control group (p = 0.0035). Survival rates were not significantly different between the control group and any treated group at Study Day 75. (**b**) Kaplan–Meier Survival Plot for 48-Hour Intervention Groups. Time to death in days for the control group and 48 h post-exposure ceftazidime treated groups (IP administration). Treatments were administered twice daily (BID) or four times daily (QID). BID low dose = 300 mg/kg/treatment, totaling 600 mg/kg/day. BID high dose = 600 mg/kg/treatment, totaling 1200 mg/kg/day. QID low dose = 150 mg/kg/treatment, totaling 600 mg/kg/day. QID high dose = 300 mg/kg/treatment, totaling 1200 mg/kg/day. At this initiation time point, a single animal survived in the high dose QID group. Time to death in the 48 h post-exposure treatment groups (52.5–58.1 h) was similar to the control group (53.2 h).

Survival from the time of inhalational exposure of *B. pseudomallei* (strain K96243) through 60 days post-cessation of ceftazidime treatment was plotted (Fig. 1a,b). Survival proportions at each stage post-treatment were compared within each group (Table 2), and between the control group and each treated group (see supplemental data Tables S1–S3). In the 24 h high dose (600 mg/kg) BID dose group, the survival rate at end of study was significantly less than at end of treatment and at 30 days post-cessation of treatment (Day 45) (p = 0.0001 and 0.0017, respectively). Survival rates in the 24 h low dose (150 mg/kg) QID dose group, the 24 h high dose BID group, and the 24 h high dose (300 mg/kg) QID group were significantly greater than that of the saline control group at the end of treatment (p = 0.0383, 0.0002, and 0.0002, respectively). At Study Day 45, survival rates of the 24 h high dose BID and 24 h high dose QID groups were significantly greater than that of the saline control group (p = 0.0035). Survival rates were not significantly different between any of the treated groups and the saline control group at the end of study (60 days post-cessation of treatment) (p values ranged from 0.1688 to 1.0000). However, times to death in the 24 h BID and QID dose groups were significantly greater than that of the saline control group (p ≤ 0.0001) (Table 3). Average time to death in the saline control group was 53.2 h. Average time to death in the 48 h treatment initiation groups was similar to the saline control group, ranging from 52.5 h to 58.1 h post-exposure.

**Table 2:** P-values from Boschloo’s tests comparing survival proportions between each pair of timepoints within each group.

Group	Group description	P-value^#^		
		End of treatment vs day 45	End of treatment vs end of study	Day 45 vs end of study
2	Saline control	1.0000	1.0000	1.0000
3	24 h low BID	1.0000	1.0000	1.0000
4	24 h high BID	0.3296	0.0001*	0.0017*
5	48 h low BID	1.0000	1.0000	1.0000
6	48 h high BID	1.0000	1.0000	1.0000
7	24 h low QID	1.0000	0.5920	0.8011
8	24 h high QID	0.5264	0.0683	0.5264
9	48 h low QID	1.0000	1.0000	1.0000
10	48 h high QID	1.0000	1.0000	1.0000

Statistical comparison of survival proportions within the control and each treated group at specific stages post-treatment including the end of treatment, 30 days post-cessation of treatment (Day 45), and the end of study. The survival rate of the 24 h high dose BID treated group was significantly less at the end of study compared to the end of treatment and Day 45, illustrating twice daily treatment likely does not meet the required threshold of %*f*T > *4MIC to adequately clear infection as significant changes in survival rate were not observed in the low dose and high dose QID treated groups at the same time points.

^#^P-value is for two-sided Boschloo’s test comparing survival rates between each pair of timepoints within each group. Overall error rate of the three tests is controlled at 5% using the Bonferroni-Holm multiple comparison procedure.

*Survival rates are significantly different between the timepoints.

**Table 3:** Kaplan–Meier median times to death with 95% confidence intervals and P-values from log rank tests comparing times to death between each of the treated groups and the vehicle group.

Group	Group description	No. died/no. in group	Kaplan–Meier median time to death in hours (95% confidence interval)	P-value^#^
2	Saline control	10/10	53.19 (51.15, 57.55)	
3	24 h low BID	10/10	140.17 (77.55, 142.07)	< 0.0001*
4	24 h high BID	10/10	1400.62 (141.02, 1441.08)	< 0.0001*
5	48 h low BID	12/12	57.09 (53.68, 60.62)	0.1495
6	48 h high BID	12/12	54.23 (52.03, 58.32)	0.6807
7	24 h low QID	8/10	356.31 (100.38, 1659.73)	< 0.0001*
8	24 h high QID	6/10	1630.67 (81.28, –)	< 0.0001*
9	48 h low QID	12/12	58.05 (53.6, 67.58)	0.0649
10	48 h high QID	11/12	52.51 (51.43, 58.68)	0.8627

The median time to death of the control group and each treated group and the statistical comparison of the treated groups to the control group. Times to death of the four 48 h post-exposure treatment initiation groups were not significantly different from the control group, indicating that 48 h is too late to therapeutically intervene at the targeted exposure dose. Times to death of the four 24 h post-exposure treatment initiation groups were significantly greater than the control group.

– Upper confidence bound could not be estimated due to large number of surviving animals.

^#^P-value is for log rank test comparing survival rates between the treated group and the saline control group. Overall error rate of the eight tests is controlled at 5% using the Bonferroni-Holm multiple comparison procedure.

*Time to death of the treated group is significantly greater than that of the control group.

### Bacterial tissue burden and bacteremia of untreated mice

**Figure 2:**
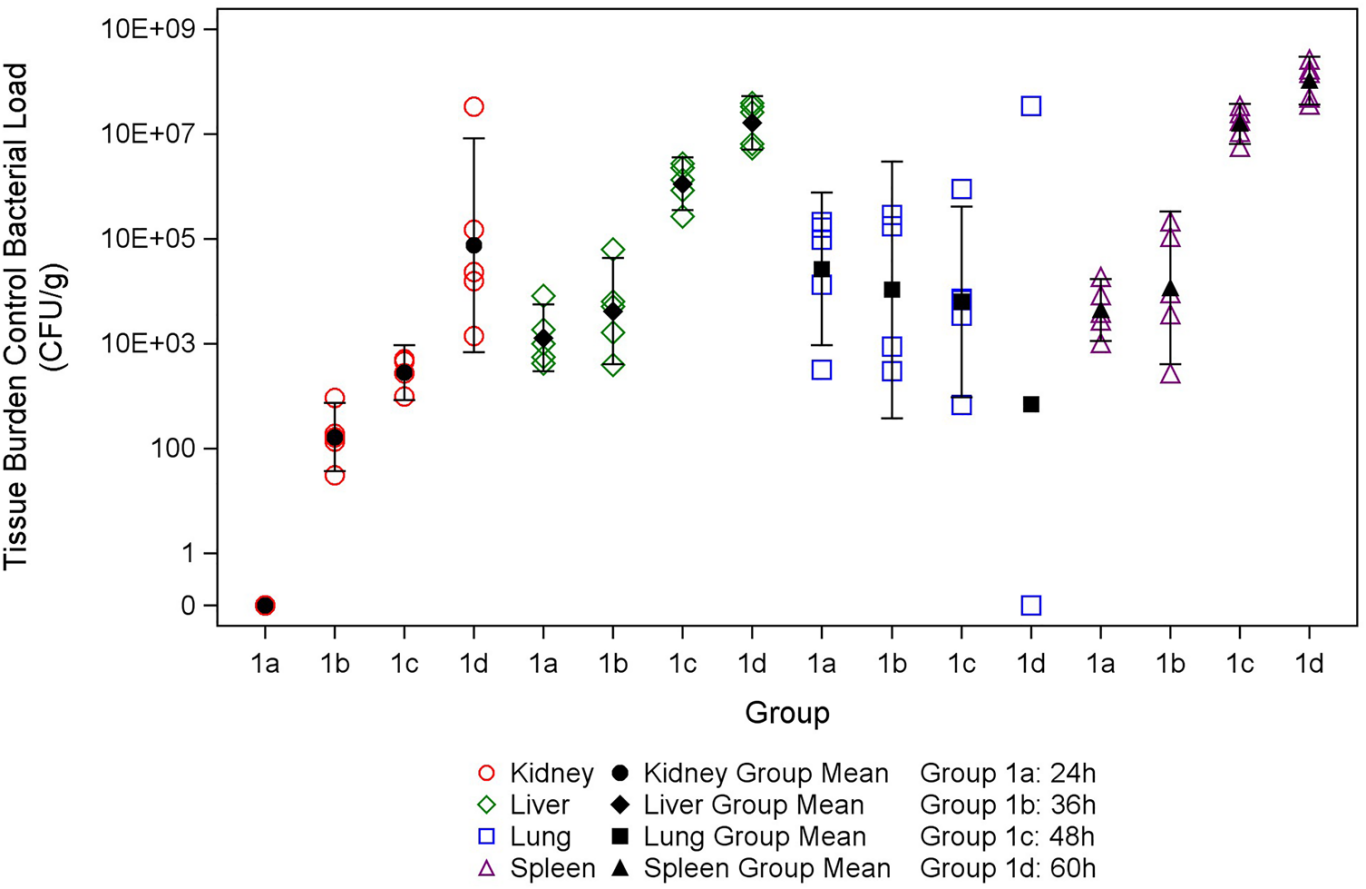
Observed bacterial load data (CFU/g) with mean and 95% confidence interval in untreated animals. Individual, mean, and 95% confidence intervals of CFU/g in the kidney, liver, lung, and spleen of untreated infected animals at 24, 36, 48, and 60 h post-exposure. All tissue specimens contained *B. pseudomallei* apart from three kidney samples at 24 h post-exposure and two lung samples at 60 h post-exposure. CFU/g in the kidney samples obtained at 36 h post-exposure was less than the lower limit of quantitation of the assay.

To characterize disease progression and assess therapeutic treatment timing at the targeted challenge dose, bacteremia and bacterial burdens in the kidney, liver, lung, and spleen were assessed in untreated mice at 24, 36, 48 and 60 h post-exposure (five mice/time point). All animals survived to scheduled collection time points apart from four animals at 60 h post-exposure. All collected blood specimens (17/17) were positive for *B. pseudomallei* from 24 through 60 h post-exposure. Blood specimens were unable to be collected from three 60 h post-exposure animals. All tissues were positive for *B. pseudomallei* apart from three kidney samples at 24 h post-exposure, and two lung samples at 60 h post-exposure. Mean bacterial loads in the liver and spleen increased at 60 h post-exposure compared to 24 h post-exposure, while mean bacterial loads in the lung decreased (Fig. 2, Table 4).

**Table 4:** Geometric means with 95% confidence intervals for tissue bacterial load in untreated animals.

Group description	Tissue	N	Geometric mean (95% confidence interval) (CFU/g)
24 h post-exposure	Kidney	3	0.00E+00 (–)
36 h post-exposure		0	NA
48 h post-exposure		4	2.82E+03 (8.55E+02, 9.28E+03)
60 h post-exposure		5	7.59E+05 (6.85E+03, 8.40E+07)
24 h post-exposure	Liver	5	1.28E+04 (2.95E+03, 5.56E+04)
36 h post-exposure		5	4.19E+04 (4.07E+03, 4.32E+05)
48 h post-exposure		5	1.13E+07 (3.57E+06, 3.57E+07)
60 h post-exposure		5	1.64E+08 (5.04E+07, 5.31E+08)
24 h post-exposure	Lung	5	2.67E+05 (9.25E+03, 7.72E+06)
36 h post-exposure		4	1.07E+05 (3.82E+02, 2.98E+07)
48 h post-exposure		5	6.35E+04 (9.65E+02, 4.18E+06)
60 h post-exposure		3	7.03E+02 (3.96E−10, 1.25E+15)
24 h post-exposure	Spleen	5	4.44E+04 (1.13E+04, 1.74E+05)
36 h post-exposure		5	1.16E+05 (4.09E+03, 3.30E+06)
48 h post-exposure		5	1.57E+08 (6.45E+07, 3.80E+08)
60 h post-exposure		5	1.04E+09 (3.63E+08, 2.96E+09)

The geometric means in CFU/g for kidney, liver, lung, and spleen bacterial burdens of infected, untreated animals at 24, 36, 48, and 60 h post-exposure. Mean bacterial burdens in the liver and spleen increased at 60 h post-exposure compared to 24 h post-exposure, while mean bacterial loads in the lung decreased.

– All measurements were the same (negative), therefore the 95% confidence interval could not be calculated.

NA No quantitative data were available.

### Bacterial tissue burden of ceftazidime treated mice

**Figure 3:**
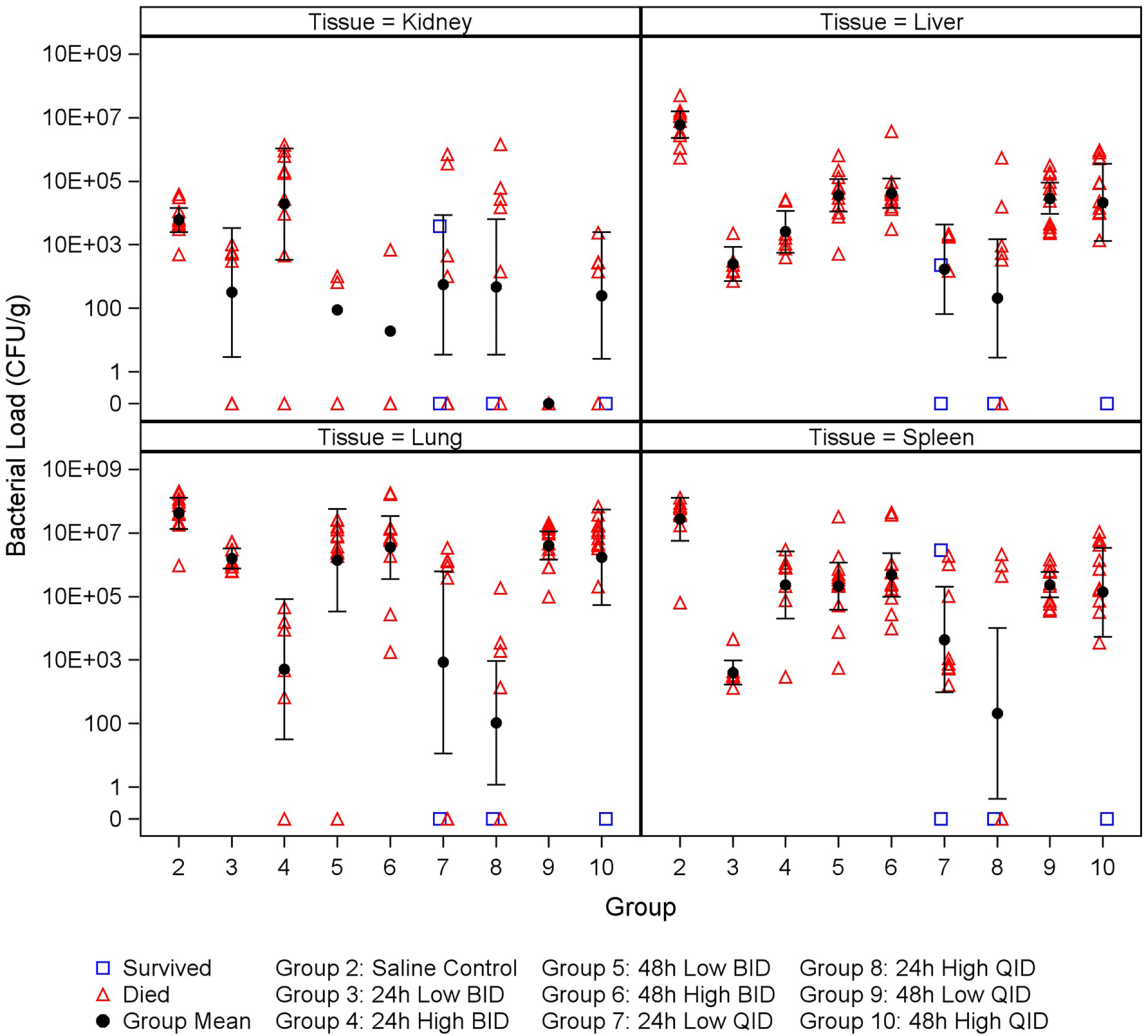
Observed bacterial load data (CFU/g) with mean and 95% confidence interval by group. Individual, mean, and 95% confidence intervals of CFU/g in the kidney, liver, lung, and spleen of infected treated animals. Specimens were collected at the time of termination or death. The mean bacterial load of the 48 h Low QID group, in which all measurements were negative, was significantly less than that of the saline group (p = 0.0127). The mean bacterial loads of all treated groups were significantly less than that of the saline group for the liver (p values ranged from < 0.0001 to 0.0024). The mean bacterial loads of the 24 h QID groups were significantly less than that of the saline group for the lung and spleen (p ≤ 0.0001). Furthermore, the mean bacterial load of the 24 h High BID group was significantly less than that of the saline group for the lung (p = 0.0001), and the mean bacterial loads of the Low BID groups and the 48 h High QID group were significantly less than that of the saline group for spleen (p values ranged from < 0.0001 to 0.0373).

Bacterial burdens in the kidney, liver, lung, and spleen were assessed in all mice that succumbed to disease or were humanely euthanized to verify presence and quantity of *B. pseudomallei* (Fig. 3, supplemental data Tables S5–S8). The mean bacterial load of only the 48 h low QID dose group, in which all measurements were negative, was significantly less than that of the saline group for the kidney (p = 0.0127). The mean bacterial loads of all treated groups were significantly less than that of the saline control group for the liver (p values ranged from < 0.0001 to 0.0024). The mean bacterial loads of the 24 h QID dose groups were significantly less than that of the saline control group for the lung and spleen (p ≤ 0.0001). Furthermore, the mean bacterial load of the 24 h high dose BID group was significantly less than that of the saline control group for the lung (p = 0.0001), and the mean bacterial loads of the low BID dose groups and the 48 h high dose QID group were significantly less than that of the saline control group for spleen (p values ranged from < 0.0001 to 0.0373).

### Murine pharmacokinetics

**Figure 4:**
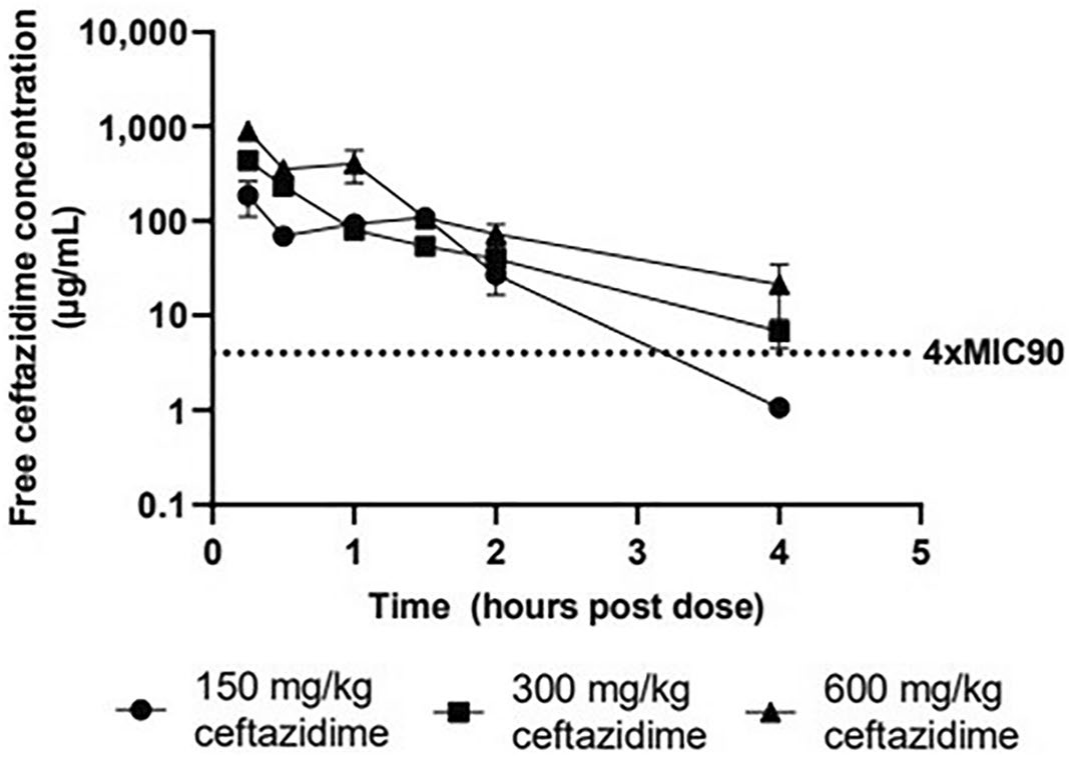
Free concentration–time curve for ceftazidime in murine plasma. The concentration–time curve of ceftazidime in murine plasma following a single dose administered IP 24 h following exposure to a lethal dose of *B. pseudomallei*. Each of the three doses administered showed a monophasic decline. Terminal phase half-lives were similar for the 300 and 600 mg/kg groups, but lower for the 150 mg/kg group. C_max_ and AUC values increased in a near dose-proportional manner for all 3 groups suggesting no dose effects on the systemic exposure of ceftazidime.

The shape of the ceftazidime plasma concentration–time curves of the three doses administered (Fig. 4) showed an apparent monophasic decline following a single administration of ceftazidime. All three dose groups had a T_max_ at the first target sample collection time of 0.25 h (Table 5). Terminal phase half-lives were similar for the 300 and 600 mg/kg groups (0.836 and 1.08 h), but lower for the 150 mg/kg group (0.389 h). While apparent clearance was similar for all three dose groups, the 150 mg/kg group had a lower apparent volume of distribution than the other two groups. When comparing the three dose levels, both C_max_ and AUC values increased in a near dose-proportional manner from 150 to 600 mg/kg, suggesting no dose effects on the systemic exposure of ceftazidime. At 150 mg/kg a mean *f*C_max_ of 251 μg/mL was observed. For a twofold and fourfold increase in dose, a 2.36-fold (592 μg/mL) and 4.86-fold (1220 μg/mL) increase in mean *f*C_max_ was observed. At 150 mg/kg a mean *f*AUC_inf_ of 281 h*μg/mL was observed. For a twofold and fourfold increase in dose, a 1.55-fold (436 h*μg/mL) and 3.67-fold (1030 h*μg/mL) increase in mean *f*AUC_inf_ was observed.

**Table 5:** Murine pharmacokinetic parameters.

IP dose (mg/kg)	Tau (h)	*f*C_max_ (µg/mL)	T_max_ (h)	Elimination half-life (h)	Elimination rate constant (h^-1^)	Apparent clearance (mL/h/kg)	Apparent volume of distribution (mL/kg)	*f*AUC_last_ (h µg/mL)	*f*AUC_∞_ (hr*µg/mL)	*f*T > 4xMIC (%)
150	6	251	0.250	0.389	1.78	532	298	281	282	69.8
300	6	592	0.304	0.836	0.829	689	830	424	436	87.2
300	12									43.7
600	12	1220	0.250	1.08	0.640	584	912	983	1030	52.3

The pharmacokinetic parameters of a single dose of ceftazidime at 150 mg/kg, 300 mg/kg, and 600 mg/kg 24 h following aerosol exposure to *B. pseudomallei*. QID dosing regimens resulted in greater PK/PD drivers of 87.2% and 69.8% *f*T > 4 µg/mL for the 300 and 150 mg/kg QID regimens, respectively. Lower PK/PD drivers of 52.3% and 43.7% *f*T > 4 µg/mL were estimated for the 600 and 300 mg/kg BID dosing regimens, respectively.

*IP* intraperitoneal.

### Clinical pharmacokinetic comparison

**Figure 5:**
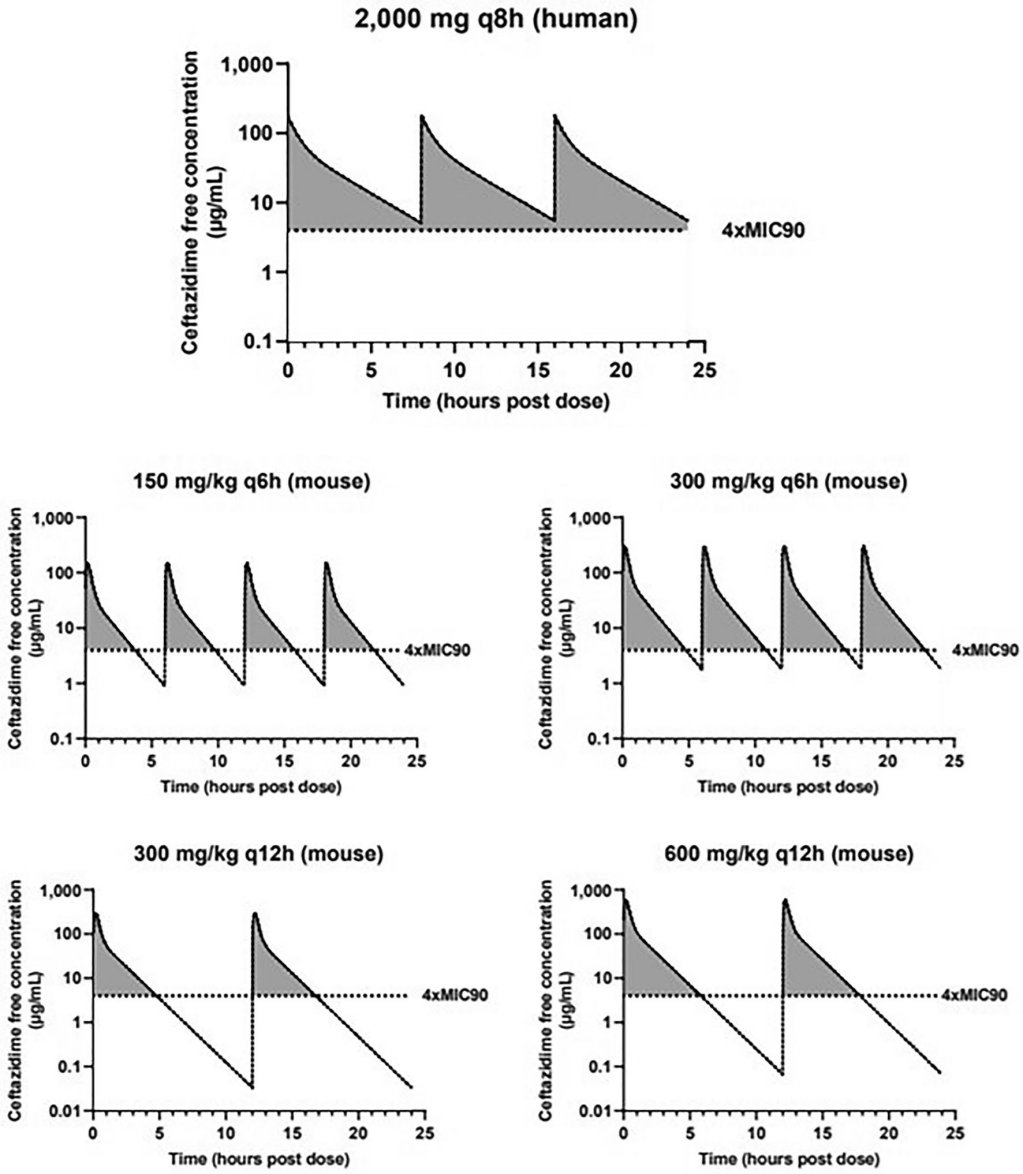
Predicted concentration–time profiles for murine and human dosing regimens. Predicted concentration–time profiles for murine and human ceftazidime dosing regimens, modeled using a PK/PD driver of 100% *f*T > 4*MIC. The clinical dose of 2000 mg (IV infusion) of ceftazidime every 8 h resulted in an estimated 100% *f*T > 4 μg/mL. In the infected murine model greater PK/PD drivers of 87.2% and 69.8% *f*T > 4 μg/mL were estimated for 300 mg/kg and 150 mg/kg QID regimens, respectively compared to lower PK/PD drivers of 52.3% and 43.7% *f*T > 4 μg/mL estimated for the 600 mg/kg and 300 mg/kg BID dosing regimens, respectively.

In comparison to a clinical acute phase standard of care dose of 2000 mg provided via IV infusion^7^, the mice that received a single 150 mg/kg intraperitoneal (IP) dose compared favorably with a *f*C_max_ of 251 µg/mL and an *f*AUC_inf_ of 282 h µg/mL compared to 129 µg/mL and 244 h μg/mL in healthy adult males^37^. Elimination half-life in mice from this study ranged from 0.389 to 1.08 h. PK data in patients showed a longer half-life of 1.9 h^37^. Using a MIC of 1 μg/mL for this strain of *B. pseudomallei*, *f*T > 4*MIC were calculated (Table 5). As anticipated the QID dosing regimens resulted in greater PK/PD drivers of 87.2% and 69.8% *f*T > 4 μg/mL (i.e., *f*T > 4*MIC) for the 300 and 150 mg/kg QID regimens, respectively. Lower PK/PD drivers of 52.3% and 43.7% *f*T > 4 µg/mL were estimated for the 600 and 300 mg/kg BID dosing regimens, respectively. The 2000 mg IV infusion of ceftazidime every 8 h resulted in an estimated 100% *f*T > 4 μg/mL which exceeds the 300 mg/kg QID regimen in mice. Predicted concentration–time profiles and the clinical data are plotted (Fig. 5) where shaded regions represent the *f*AUC for the portion of the profile that exceeds 4 μg/mL.

## Discussion

Several murine melioidosis models have been described in the literature with the objective of mimicking known routes of exposure and disease manifestation in human populations. In regard to biodefense research, the inhalation route is thought to be a likely route of exposure in a deliberate release of *B. pseudomallei* with the intent to infect a large population with a high concentration of agent. *B. pseudomallei* strain K96243 is commonly used in the laboratory setting and aerosolization of this strain to BALB/c mice has been described^15,19,21^ and has also been previously characterized in our laboratory (data not published). MCM evaluations have described targeted challenge doses of 100 MLD or 100 × LD_50_^21,26^. Thomas et al*.* describe a lower infective dose at 100 MLD (627 cfu) versus the 69 × LD_50_ (11,100 cfu) dose in the current study, however, the median time to death of untreated controls was similar in both studies (2.5 and 2.2 days, respectively).

A cohort of untreated animals was serially sacrificed at 24, 36, 48, and 60 h post-exposure to confirm that treatment initiation at 24 h was therapeutic intervention. All animals were bacteremic and bacterial loads were present in three of four assessed tissues by 24 h. At 48 h, tissue burdens in the kidney, liver, and spleen were 3 to 4 logs greater compared to 24 h (Table 4). Time to death in the 48 h post-exposure initiation treatment groups ranged from 52.5 to 58.1 h and was not significantly different from the control group (53.2 h), indicating 48 h is too late to intervene in the murine model at the targeted exposure dose. In studies where *B. pseudomallei* strain K96243 was aerosolized at a considerably lower infective dose (3.8–5 cfu) delayed bacteremia and slightly delayed tissue dissemination and time to death was observed^15,19^ compared with the current study.

Clinically, melioidosis most commonly presents with pneumonia^38^. Pneumonia may result in septic shock carrying a high mortality rate or may be mild in nature with a low mortality rate^8^. In Meumann, et al.’s review of the Royal Darwin Hospital’s database from 1989 through 2010^39^, 20% of patients with primary pneumonia died (median time to death of three days) with several patients found dead upon arrival to the hospital, dying on the day of admission, or dying the day following admission. While a mouse model of a low infective dose may provide a similar disease progression to many clinical melioidosis presentations and outcomes, the higher infective dose and expedited disease progression illustrated in this study and other MCM evaluations previously noted allow for a more stringent MCM evaluation model for comparison to the clinically acute cases that result in death within a shorter time frame and provide for therapies that are more likely to be effective in both severe and mild disease states.

Treatment of melioidosis is divided into acute and eradication phases. For the acute phase, ceftazidime may be administered IV for at least two weeks^6^. *B. pseudomallei* resistance to ceftazidime has been documented^5,40^, necessitating the identification of new effective acute phase treatments or strategies that eliminate biphasic treatment. As stated previously, the PK/PD driver for cephalosporins such as ceftazidime is *f*T > MIC and the optimal clinical target for *P. aeruginosa* has been reported as 100% *f*T > 4*MIC, even though this target was not readily applicable to severe infections of *B pseudomallei*^13^. Since the pharmacokinetics could not readily be recapitulated in mice, four IP dosing regimens were examined that were intended to result in a range of exposures. The dosing regimen was also varied to increase the *f*T > MIC given the rapid clearance of ceftazidime in mice. Dosing regimens of 300 and 600 mg/kg administered BID were intended to result in a lower *f*T > 4*MIC and lead to a suboptimal treatment. Dosing regimens of 150 or 300 mg/kg administered QID were intended to result in greater *f*T > 4*MIC that are more comparable to an approved clinical dosing regimen of ceftazidime (two grams, three times daily by intravenous infusion^37^), which should result in better survival outcomes. From previous data obtained in our laboratory, doses of 600 or 1200 mg/kg/day when administered BID resulted in statistically significant increases in survival through 21 days post-treatment cessation compared to saline control animals (data not published). In the current study, these same doses were utilized in addition to administering the same daily doses over six hour intervals to fully characterize the disease model. Two treatment initiation time points were studied: 24 h and 48 h post-exposure, totaling eight distinct treatment regimens. Because the 48 h post-exposure mice succumbed to disease shortly after treatment was initiated, further discussion regarding efficacy outcomes herein is limited to the four 24 h intervention groups. While a humanized ceftazidime dose was not expected to be achieved in this study, a favorable %*f*T > 4*MIC was only achieved in the 300 mg/kg QID dose. One clinical study observed 41% survival following treatment with 4.8–6.0 g/day ceftazidime in septic and/or bacteremic patients^13^. In the current study, better or comparable survival at the end of the treatment period was observed when ceftazidime treatment was initiated 24 h following exposure and administered at 600 mg/kg BID (90%), 300 mg/kg QID (90%) or 150 mg/kg QID (50%). No animals survived through the end of the treatment period in the low dose BID group. Short term survival in the high dose BID group outperformed the low dose QID group, even though the PK/PD modeling provided for slightly higher *f*T > 4*MIC percentage in the low dose QID group (52.3% high dose BID versus 69.8%, low dose QID). By the end of the study period, survival rates in the two groups were not significantly different, suggesting that both dosages may only have been high enough to achieve bacteriostasis. Additional mortality occurred within 30 days of cessation of treatment in each of the three surviving groups, resulting in survival rates of 70% in both high dose groups and 40% in the low dose QID group. By 60 days post-cessation of treatment, all mice in the high dose BID group succumbed to disease, while 20% and 40% of mice in the low dose QID and high dose QID groups survived, respectively. The low dose QID survival rate is similar to another study where ceftazidime treatment was initiated 24 h following inhalation exposure to a targeted 100 × LD_50_ dose of strain 1026b and resulted in 20% survival 60 days post-cessation of treatment^26^. The therapeutic regimen described in McCurdy, et al*.* was similar to the current study regarding dosage, dose route, and dose frequency, with the duration of treatment being extended a week compared with the current study. Efficacy evaluations including ceftazidime have been described in the murine model with varying dosing strategies. Single daily subcutaneous dosing^32^, single daily IP dosing^31^, BID IP dosing^29,30^, and QID IP dosing have all been employed. Treatment duration has varied from three days^32^ to 28 days^29^. Single ceftazidime doses ranging from 25^32^ to 1200 mg/kg^31^ have been utilized. Depending on the MCM being evaluated, varying frequencies, durations, and doses of ceftazidime may be appropriate. Murine pharmacokinetics of ceftazidime in the melioidosis model are scant in the literature; Ulett and Hirst et al. described basing ceftazidime dosage on T > MIC, however it was not described if this was based on free concentration and for what percentage of time the 12 h dosing interval remained above the MIC. This study provides PK parameters of three ceftazidime doses and subsequent PK/PD modeling of those doses which may be of assistance in informing dose selection of MCM evaluations.

Following exposure to ceftazidime, there was significant reduction in bacterial burden in the liver, lung, and spleen in both QID dose groups compared to the control group. In the high dose BID group, there was significant reduction in bacterial burden in the liver and lung. A small number of terminal blood specimens was successfully collected for qualitative bacteremia assessment. Four of five (80%) specimens in the low dose QID group were bacteremic, three of four (75%) specimens in the high dose BID group were bacteremic, and zero of four (0%) specimens in the high dose QID group were bacteremic. Five of the six blood specimens that were negative for bacterial growth correlated to negative tissue burdens in the corresponding animals. The single blood specimen that was negative for bacterial growth in the high dose BID group correlated with one negative tissue burden and three tissues that were positive for *B. pseudomallei*; however, for the positive tissue burdens, fewer than 25 colonies were present, on average. While most murine evaluations include bacterial tissue burden assessment as a component of efficacy, the length of monitoring animals post-cessation of treatment has not been consistent, which is important considering the potential for relapse in cases of melioidosis. Several references do not describe survival monitoring post-cessation of treatment^29,30,41^ while other references describe monitoring for survival up to 60 days post-cessation of treatment^26,32^. While the appropriate monitoring period may vary depending on study objectives, some length of monitoring post-cessation of treatment should be considered. The current study illustrates this point; while a high dose BID treatment regimen appeared to be protective at the end of the treatment regimen, no survivors in the group remained 60 days post-cessation of treatment.

The “2010 Workshop on Treatment of and Postexposure Prophylaxis for *B. pseudomallei* and *B. mallei*”^14^ concluded that there is a need to standardize animal studies of MCM evaluations. For biodefense preparedness evaluations, a 1200 mg/kg/day dose of ceftazidime administered approximately every six hours at 300 mg/kg in mice provides protection through the acute phase of melioidosis at a targeted infective dose of 50 × LD_50_. However, this dose does not prevent recrudescence. In evaluations of novel MCMs, this is an adequate acute phase dose to utilize as a comparator antibiotic or it could potentially be used to evaluate ceftazidime as part of a combination therapy.

## Supplementary Information

Supplementary Information.

## Data Availability

The data that support the findings of this study are available from Battelle, but restrictions apply to the availability of these data, which were used under license for the current study, and so are not publicly available. Data are however available from the authors upon reasonable request and with permission of Battelle.
