# Supplementary material for: Efficacy of ceftazidime in a murine model following a lethal aerosol exposure to Burkholderia pseudomallei
Source: Sci Rep. 2023 Mar 10;13:4047. doi: 10.1038/s41598-023-31131-8 (PMC10006082; doi:10.1038/s41598-023-31131-8)
Supplement: Supplementary file 1 — Supplementary Information. [file 41598_2023_31131_MOESM1_ESM.docx]

Supplementary Material - Efficacy of Ceftazidime in a Murine Model Following a Lethal Aerosol Exposure to *Burkholderia pseudomallei*

**Table S1. Survival Rates with Corresponding 95% Binomial Confidence Intervals and P-Values from Boschloo’s Tests Comparing Survival Proportions Between Each of the Treated Groups and the Saline Control Group at End of Treatment.**

| **Group** | **Group Description** | **No. Survived /**  **No. in Group** | **Survival Proportion**  **(95% Confidence Interval)** | **P-Value^#^** |
| --- | --- | --- | --- | --- |
| 2 | Saline Control | 0 / 10 | 0.00 (0.00, 0.31) |  |
| 3 | 24h Low BID | 0 / 10 | 0.00 (0.00, 0.31) | 1.0000 |
| 4 | 24h High BID | 9 / 10 | 0.90 (0.55, 1.00) | 0.0002* |
| 5 | 48h Low BID | 0 / 12 | 0.00 (0.00, 0.26) | 1.0000 |
| 6 | 48h High BID | 0 / 12 | 0.00 (0.00, 0.26) | 1.0000 |
| 7 | 24h Low QID | 5 / 10 | 0.50 (0.19, 0.81) | 0.0383* |
| 8 | 24h High QID | 9 / 10 | 0.90 (0.55, 1.00) | 0.0002* |
| 9 | 48h Low QID | 0 / 12 | 0.00 (0.00, 0.26) | 1.0000 |
| 10 | 48h High QID | 1 / 12 | 0.08 (0.00, 0.38) | 1.0000 |

# P-value is for one-sided Boschloo’s test comparing survival rates between the treated group and the saline control group. Overall error rate of the eight tests is controlled at 5% using the Bonferroni-Holm multiple comparison procedure.

* Survival rate of the treated group is significantly greater than that of the control group.

Table S1 illustrates the statistical comparison of survival proportions of each treated group to the control group at the end of the treatment period. The 24h high dose BID treatment group, and the 24h QID treatment groups had a significantly greater rate of survival at the end of ceftazidime treatment compared to the control group.

**Table S2. Survival Rates with Corresponding 95% Binomial Confidence Intervals and P-Values from Boschloo’s Tests Comparing Survival Proportions Between Each of the Treated Groups and the Saline Control Group at Day 45.**

| **Group** | **Group Description** | **No. Survived /**  **No. in Group** | **Survival Proportion**  **(95% Confidence Interval)** | **P-Value^#^** |
| --- | --- | --- | --- | --- |
| 2 | Saline Control | 0 / 10 | 0.00 (0.00, 0.31) |  |
| 3 | 24h Low BID | 0 / 10 | 0.00 (0.00, 0.31) | 1.0000 |
| 4 | 24h High BID | 7 / 10 | 0.70 (0.35, 0.93) | 0.0035* |
| 5 | 48h Low BID | 0 / 12 | 0.00 (0.00, 0.26) | 1.0000 |
| 6 | 48h High BID | 0 / 12 | 0.00 (0.00, 0.26) | 1.0000 |
| 7 | 24h Low QID | 4 / 10 | 0.40 (0.12, 0.74) | 0.1266 |
| 8 | 24h High QID | 7 / 10 | 0.70 (0.35, 0.93) | 0.0035* |
| 9 | 48h Low QID | 0 / 12 | 0.00 (0.00, 0.26) | 1.0000 |
| 10 | 48h High QID | 1 / 12 | 0.08 (0.00, 0.38) | 1.0000 |

# P-value is for one-sided Boschloo’s test comparing survival rates between the treated group and the saline control group. Overall error rate of the eight tests is controlled at 5% using the Bonferroni-Holm multiple comparison procedure.

* Survival rate of the treated group is significantly greater than that of the control group.

Table S2 illustrates the statistical comparison of survival proportions of each treated group to the control group 30 days post-cessation of ceftazidime treatment (Day 45). The 24h high dose BID treatment group, and the 24h high dose QID treatment group had a significantly greater rate of survival at Day 45 compared to the control group.

**Table S3. Survival Rates with Corresponding 95% Binomial Confidence Intervals and P-Values from Boschloo’s Tests Comparing Survival Proportions Between Each of the Treated Groups and the Saline Control Group at End of Study.**

| **Group** | **Group Description** | **No. Survived /**  **No. in Group** | **Survival Proportion**  **(95% Confidence Interval)** | **P-Value^#^** |
| --- | --- | --- | --- | --- |
| 2 | Saline Control | 0 / 10 | 0.00 (0.00, 0.31) |  |
| 3 | 24h Low BID | 0 / 10 | 0.00 (0.00, 0.31) | 1.0000 |
| 4 | 24h High BID | 0 / 10 | 0.00 (0.00, 0.31) | 1.0000 |
| 5 | 48h Low BID | 0 / 12 | 0.00 (0.00, 0.26) | 1.0000 |
| 6 | 48h High BID | 0 / 12 | 0.00 (0.00, 0.26) | 1.0000 |
| 7 | 24h Low QID | 2 / 10 | 0.20 (0.03, 0.56) | 0.9217 |
| 8 | 24h High QID | 4 / 10 | 0.40 (0.12, 0.74) | 0.1688 |
| 9 | 48h Low QID | 0 / 12 | 0.00 (0.00, 0.26) | 1.0000 |
| 10 | 48h High QID | 1 / 12 | 0.08 (0.00, 0.38) | 1.0000 |

# P-value is for one-sided Boschloo’s test comparing survival rates between the treated group and the saline control group. Overall error rate of the eight tests is controlled at 5% using the Bonferroni-Holm multiple comparison procedure. There were no significant differences in survival between any of the treated groups and the control group.

Table S3 illustrates the statistical comparison of survival proportions of each treated group to the control group at the end of study. There were no significant differences in survival between any treated group and the control group 60 days post-cessation of ceftazidime treatment.

**Table S4. Proportion Positive for Bacteremia with Corresponding 95% Binomial Confidence Intervals.**

| **Group** | **Group Description** | **No. Positive /**  **No. in Group** | **Proportion Positive**  **(95% Confidence Interval)** | **P-Value^#^** |
| --- | --- | --- | --- | --- |
| 1 | Tissue Burden Control | 17 / 17 | 1.00 (0.80, 1.00) |  |
| 3 | 24h Low BID | 3 / 3 | 1.00 (0.29, 1.00) | NA |
| 4 | 24h High BID | 3 / 4 | 0.75 (0.19, 0.99) | NA |
| 5 | 48h Low BID | 1 / 1 | 1.00 (0.03, 1.00) | NA |
| 6 | 48h High BID | 1 / 1 | 1.00 (0.03, 1.00) | NA |
| 7 | 24h Low QID | 4 / 5 | 0.80 (0.28, 0.99) | NA |
| 8 | 24h High QID | 0 / 4 | 0.00 (0.00, 0.60) | NA |
| 9 | 48h Low QID | 1 / 1 | 1.00 (0.03, 1.00) | NA |
| 10 | 48h High QID | 0 / 1 | 0.00 (0.00, 0.98) | NA |

# No comparisons were performed because data was not available for the saline group.

Bacteremia data not collected for animals found dead, only at euthanasia.

Table S4 illustrates the proportion of animals in the control and each treated group that were bacteremic at time of death. Five of the six negative bacteremia specimens correlated to negative tissue burdens in the corresponding animals. The negative bacteremia specimen in the 24h high dose BID group correlated with one negative tissue burden and three tissues that were positive for *B. pseudomallei*; however, for the positive tissue burdens, fewer than 25 colonies were present, on average.

**Table S5. Geometric Means with 95% Confidence Intervals for Bacterial Load in Kidney and Dunnett-Adjusted P-Values Comparing Mean Bacterial Load Between Each of the Treated Groups and the Saline Control Group.**

| **Group** | **Group Description** | **N** | **Geometric Mean**  **(95% Confidence Interval)**  **(CFU/g)** | **P-Value^#^** |
| --- | --- | --- | --- | --- |
| 1a | 24h Tissue Burden Control | 3 | 0.00E+00 (--) |  |
| 1b | 36h Tissue Burden Control | 0 | NA |  |
| 1c | 48h Tissue Burden Control | 4 | 2.82E+03 (8.55E+02, 9.28E+03) |  |
| 1d | 60h Tissue Burden Control | 5 | 7.59E+05 (6.85E+03, 8.40E+07) |  |
| 2 | Saline Control | 10 | 6.11E+04 (2.54E+04, 1.47E+05) |  |
| 3 | 24h Low BID | 6 | 3.14E+02 (2.88E+00, 3.43E+04) | 0.2753 |
| 4 | 24h High BID | 9 | 1.90E+05 (3.42E+03, 1.06E+07) | 0.9989 |
| 5 | 48h Low BID | 3 | 8.83E+01 (5.66E-03, 1.38E+06) | 0.3033 |
| 6 | 48h High BID | 3 | 1.90E+01 (5.94E-05, 6.10E+06) | 0.1204 |
| 7 | 24h Low QID | 9 | 5.48E+02 (3.43E+00, 8.75E+04) | 0.2699 |
| 8 | 24h High QID | 10 | 4.69E+02 (3.49E+00, 6.29E+04) | 0.2128 |
| 9 | 48h Low QID | 3 | 0.00E+00 (--) | 0.0127* |
| 10 | 48h High QID | 6 | 2.51E+02 (2.52E+00, 2.51E+04) | 0.2344 |

-- All measurements were the same (negative), therefore the 95% confidence interval could not be calculated.

# P-value is for the T-test comparing mean bacterial load of the treated group and the saline control group. Overall error rate of the eight tests is controlled at 5% using Dunnett’s multiple comparison procedure.

* Mean bacterial load of the treated group is significantly less than that of the saline group.

NA No quantitative data were available.

Table S5 illustrates the geometric means of the CFU/g of bacterial burden in kidney of untreated and treated groups. The treated groups were compared to the saline control group. Only the 48h low dose QID treated group had significantly less bacterial burden in the kidney compared to the control group.

**Table S6. Geometric Means with 95% Confidence Intervals for Bacterial Load in Liver and Dunnett-Adjusted P-Values Comparing Mean Bacterial Load Between Each of the Treated Groups and the Saline Control Group.**

| **Group** | **Group Description** | **N** | **Geometric Mean**  **(95% Confidence Interval)**  **(CFU/g)** | **P-Value^#^** |
| --- | --- | --- | --- | --- |
| 1a | 24h Tissue Burden Control | 5 | 1.28E+04 (2.95E+03, 5.56E+04) |  |
| 1b | 36h Tissue Burden Control | 5 | 4.19E+04 (4.07E+03, 4.32E+05) |  |
| 1c | 48h Tissue Burden Control | 5 | 1.13E+07 (3.57E+06, 3.57E+07) |  |
| 1d | 60h Tissue Burden Control | 5 | 1.64E+08 (5.04E+07, 5.31E+08) |  |
| 2 | Saline Control | 10 | 6.06E+07 (2.29E+07, 1.60E+08) |  |
| 3 | 24h Low BID | 6 | 2.53E+03 (7.30E+02, 8.75E+03) | <0.0001* |
| 4 | 24h High BID | 7 | 2.56E+04 (5.59E+03, 1.18E+05) | <0.0001* |
| 5 | 48h Low BID | 12 | 3.59E+05 (1.12E+05, 1.15E+06) | 0.0016* |
| 6 | 48h High BID | 12 | 4.24E+05 (1.47E+05, 1.23E+06) | 0.0024* |
| 7 | 24h Low QID | 7 | 1.69E+03 (6.64E+01, 4.30E+04) | <0.0001* |
| 8 | 24h High QID | 10 | 2.07E+02 (2.85E+00, 1.50E+04) | <0.0001* |
| 9 | 48h Low QID | 12 | 2.88E+05 (9.27E+04, 8.93E+05) | 0.0009* |
| 10 | 48h High QID | 12 | 2.13E+05 (1.31E+04, 3.47E+06) | 0.0004* |

# P-value is for the T-test comparing mean bacterial load of the treated group and the saline control group. Overall error rate of the nine tests is controlled at 5% using Dunnett’s multiple comparison procedure.

* Mean bacterial load of the treated group is significantly less than that of the saline group.

Table S6 illustrates the geometric means of the CFU/g of bacterial burden in liver of untreated and treated groups. The treated groups were compared to the saline control group. All treated groups had significantly less bacterial burden in the liver compared to the control group.

**Table S7. Geometric Means with 95% Confidence Intervals for Bacterial Load in Lung and Dunnett-Adjusted P-Values Comparing Mean Bacterial Load Between Each of the Treated Groups and the Saline Control Group.**

| **Group** | **Group Description** | **N** | **Geometric Mean**  **(95% Confidence Interval)**  **(CFU/g)** | **P-Value^#^** |
| --- | --- | --- | --- | --- |
| 1a | 24h Tissue Burden Control | 5 | 2.67E+05 (9.25E+03, 7.72E+06) |  |
| 1b | 36h Tissue Burden Control | 4 | 1.07E+05 (3.82E+02, 2.98E+07) |  |
| 1c | 48h Tissue Burden Control | 5 | 6.35E+04 (9.65E+02, 4.18E+06) |  |
| 1d | 60h Tissue Burden Control | 3 | 7.03E+02 (3.96E-10, 1.25E+15) |  |
| 2 | Saline Control | 10 | 4.21E+08 (1.36E+08, 1.30E+09) |  |
| 3 | 24h Low BID | 8 | 1.57E+07 (7.50E+06, 3.30E+07) | 0.5822 |
| 4 | 24h High BID | 6 | 5.20E+03 (3.22E+01, 8.39E+05) | 0.0001* |
| 5 | 48h Low BID | 11 | 1.39E+07 (3.37E+05, 5.72E+08) | 0.4509 |
| 6 | 48h High BID | 11 | 3.49E+07 (3.49E+06, 3.48E+08) | 0.7697 |
| 7 | 24h Low QID | 9 | 8.46E+03 (1.14E+01, 6.27E+06) | <0.0001* |
| 8 | 24h High QID | 9 | 1.06E+02 (1.20E+00, 9.32E+03) | <0.0001* |
| 9 | 48h Low QID | 12 | 4.00E+07 (1.43E+07, 1.12E+08) | 0.7983 |
| 10 | 48h High QID | 12 | 1.74E+07 (5.46E+05, 5.56E+08) | 0.5050 |

# P-value is for the T-test comparing mean bacterial load of the treated group and the saline control group. Overall error rate of the nine tests is controlled at 5% using Dunnett’s multiple comparison procedure.

* Mean bacterial load of the treated group is significantly less than that of the saline group.

Table S7 illustrates the geometric means of the CFU/g of bacterial burden in lung of untreated and treated groups. The treated groups were compared to the saline control group. The 24h high dose BID treated group and 24h QID treated groups had significantly less bacterial burden in the lung compared to the control group.

**Table S8. Geometric Means with 95% Confidence Intervals for Bacterial Load in Spleen and Dunnett-Adjusted P-Values Comparing Mean Bacterial Load Between Each of the Treated Groups and the Saline Control Group.**

| **Group** | **Group Description** | **N** | **Geometric Mean**  **(95% Confidence Interval)**  **(CFU/g)** | **P-Value^#^** |
| --- | --- | --- | --- | --- |
| 1a | 24h Tissue Burden Control | 5 | 4.44E+04 (1.13E+04, 1.74E+05) |  |
| 1b | 36h Tissue Burden Control | 5 | 1.16E+05 (4.09E+03, 3.30E+06) |  |
| 1c | 48h Tissue Burden Control | 5 | 1.57E+08 (6.45E+07, 3.80E+08) |  |
| 1d | 60h Tissue Burden Control | 5 | 1.04E+09 (3.63E+08, 2.96E+09) |  |
| 2 | Saline Control | 10 | 2.71E+08 (5.62E+07, 1.31E+09) |  |
| 3 | 24h Low BID | 8 | 4.00E+03 (1.67E+03, 9.62E+03) | <0.0001* |
| 4 | 24h High BID | 8 | 2.35E+06 (2.05E+05, 2.68E+07) | 0.0842 |
| 5 | 48h Low BID | 12 | 2.12E+06 (3.79E+05, 1.19E+07) | 0.0373* |
| 6 | 48h High BID | 12 | 4.82E+06 (9.85E+05, 2.36E+07) | 0.1185 |
| 7 | 24h Low QID | 10 | 4.37E+04 (9.56E+02, 2.00E+06) | <0.0001* |
| 8 | 24h High QID | 9 | 2.13E+02 (4.37E-01, 1.03E+05) | <0.0001* |
| 9 | 48h Low QID | 11 | 2.35E+06 (9.49E+05, 5.83E+06) | 0.0501 |
| 10 | 48h High QID | 12 | 1.36E+06 (5.43E+04, 3.42E+07) | 0.0184* |

# P-value is for the T-test comparing mean bacterial load of the treated group and the saline control group. Overall error rate of the nine tests is controlled at 5% using Dunnett’s multiple comparison procedure.

* Mean bacterial load of the treated group is significantly less than that of the saline group.

Table S8 illustrates the geometric means of the CFU/g of bacterial burden in spleen of untreated and treated groups. The treated groups were compared to the saline control group. The 24h and 48h low dose BID treated groups, the 24h QID treated groups, and the 48h high dose QID treated group had significantly less bacterial burden in the spleen compared to the control group.
